# Altitude effect on Propofol Pharmacokinetics in Rats

**DOI:** 10.2174/0113892002285571240220131547

**Published:** 2024-03-11

**Authors:** Lijun Li, Xuejun Wang, Sheng Wang, Li Wen, Haopeng Zhang

**Affiliations:** 1 Department of Anesthesiology, The First People's Hospital of Ziyang City, Ziyang, 641300, China;; 2 Department of Anesthesiology, Qinghai Red Cross Hospital, Xining, 810000, China;; 3 Department of Anesthesiology, Dazhou Central Hospital, Dazhou, 635000, China;; 4 Department of Anesthesiology, The Third Military Medical University, Chongqing, 400000, China;; 5 Department of Anesthesiology, Xijing Hospital of Air Force Military Medical University, Xi'an, 710000, China

**Keywords:** Propofol, pharmacokinetics, high-altitude, hypoxia, gas mass spectrometry, righting flection

## Abstract

**Background:**

Propofol is an intravenous agent for clinical anesthesia. As the influence of the hypobaric-hypoxic environment (Qinghai-Tibetan region, altitude: 2800-4300 m, PaO2: 15.1-12.4 kPa) on the metabolism of Propofol is complex, the research results on the metabolic characteristics of Propofol in high-altitude areas remain unclear. This study aimed to investigate the pharmacokinetic characteristics of Propofol in a high-altitude hypoxic environment using animal experiments.

**Methods:**

Rats were randomly divided into three groups: high-altitude, medium-altitude, and plain groups. The time of disappearance and recovery of the rat righting reflex was recorded as the time of anesthesia induction and awakening, respectively. The plasma concentration of Propofol was determined by gas chromatography-mass spectrometry. A pharmacokinetic analysis software was used to analyze the blood-drug concentrations and obtain the pharmacokinetic parameters.

**Results:**

We observed that when Propofol anesthetizes rats, the anesthesia induction time was shortened, and the recovery time was prolonged with increased altitude. Compared with the plain group, the clearance of Propofol decreased, whereas the half-life, area under the concentration-time curve, peak plasma concentration, and average residence time extension increased.

**Conclusion:**

The pharmacokinetic characteristics of Propofol are significantly altered in high-altitude hypoxic environments.

## 1 INTRODUCTION

High-altitude areas have low pressure, low oxygen, low temperature, high radiation, and dry climates, which collectively adversely affect the human body. Hypoxia is one of the oldest pathogenic conditions [1, 2]. The absorption, distribution, metabolism, and excretion of drugs change in high-altitude environments, changing their pharmacokinetic parameters [3].

Currently, reports on the pharmacokinetic changes of intravenous anesthetics under hypoxic conditions are limited. Little research has been conducted on the metabolic characteristics of Propofol in high-altitude hypoxic environments. As Propofol was first used for clinical anesthesia in 1973, it has been one of the most widely used intravenous anesthetics worldwide. Propofol has the advantages of rapid onset of action, short action time, and complete recovery; compared to inhaled anesthetics, the incidence of postoperative nausea, vomiting, and delirium is lower with Propofol [4].

The effect of high-altitude environments on Propofol pharmacokinetics is complex. Studies have confirmed that simulated plateau environments completely differ from actual plateau environments. Hypoxia models induced by low-pressure cabins or chemical methods can only simulate hypoxia but not hypothermia, intense radiation, dry climate, or a real atmosphere, indicating that these methods cannot reflect a real high-altitude environment [5-8]. Furthermore, the effect of chronic hypoxia on the pharmacokinetic parameters of Propofol remains unclear.

This study aimed to investigate the pharmacokinetic changes in Propofol in a high-altitude hypoxic environment to provide a theoretical basis for the rational clinical use of Propofol in high-altitude areas. It is necessary to achieve personalized anesthesia in high-altitude regions.

## 2 MATERIALS AND METHODS

### 2.1 Animal Experiments

*Grouping and modeling:* All experiments were performed on male Sprague-Dawley specific-pathogen-free (SPF) rats (8-12 weeks old, 300-350 g) purchased from the Animal Experiment Center of Xi'an Jiaotong University with animal license number SCXK (Shanxi) 2,018-001. All experimental procedures were performed in strict accordance with the National Institutes of Health Guide for the Care and Use of Laboratory Animals. The protocol was approved by the Research Ethics Committee of Qinghai Red Cross Hospital. The approval number of the ethics committee for "Altitude effect on Propofol pharmacokinetics in rats" is KY-2018-12. All rats were maintained under temperature- (22-25°C) and humidity-controlled (55-60%) conditions, with a 12-h light/dark cycle (8:00-20:00). The rats were allowed to acclimatize to the environment for 1 month before the experiments.

Using the random-number table method, the rats were randomly divided into three groups (n=12): high-altitude hypoxic (P1; altitude: 3,900 m, PaO_2_: 12.9 kPa), moderate-altitude hypoxic (P2; altitude: 2,300 m, PaO_2_: 16.1 kPa), and plain (P3; altitude: 390 m, PaO_2_: 20 kPa) groups. The rats in the plain group were bred and kept in the city of Xi'an, located northwest of China's Shanxi Province, while those in the moderate- and high-altitude groups were transported by bus to Xining and Chengduo counties in Qinghai Province, China, respectively.

*The method of jugular vein catheterization:* The rats were anesthetized by intraperitoneal injection of 2.5% pentobarbital sodium (40 mg/kg), catheterized in their jugular and femoral veins, and an indwelling tube was fixed on their backs. After the operation, the rats were kept in a single cage and were provided sufficient food and water, and the experiment was continued after 1 week of rest.

*Blood sample collection:* The rats were placed in the transparent drum, and 50 mg_·_kg^-1^_·_h^-1^ [9, 10] of Propofol was continuously administered through the jugular vein for anesthesia. Anesthesia was maintained for 1 hour. The time of the loss of the righting reflex was recorded as the anesthesia induction time, which refers to the period starting from Propofol administration until the rat's abdomen faced up and the body cannot be turned over when the roller rotates 90° every 15 seconds. When the righting reflex disappeared, the roller was turned on to restore it to its initial state. The time of recovery of the righting reflex was recorded as awakening time, which refers to the period from the discontinuation of Propofol injection to when the rat stands on all four feet when the roller rotates 90° every 15 seconds. A 1 mL heparinized syringe was used to collect 0.3 mL of venous blood through the femoral vein at the following time points: before administration of Propofol; when the righting reflex disappeared; 2, 4, 8, 12, 20, 30, and 60 min after Propofol administration; when the righting reflex was restored; and 2, 15, 30, and 60 min after discontinuation of Propofol. The collected venous blood was centrifuged at 15,000 rpm for 12 min. The supernatant was collected, added to the cryotube, and placed in an ultra-low temperature refrigerator at -80 °C for storage.

### 2.2 Method of Measuring the Blood Concentration of Propofol

*Instruments and reagents:* Blood samples collected in this experiment were analyzed using gas chromatography/mass spectrometry (ISQ LT, Thermo Scientific, USA). Thymol (5-Methyl-2-isopropylphenol, Sigma Company, USA) was used as the Propofol standard reference substance (purity>99.8%): the internal standard.

*Chromatographic conditions*: The column thermo TG-SMS (30 m × 0.32 mm×0.25 μm) was used. High-purity nitrogen was used as the carrier gas, and the column flow rate was maintained at 1.0 m/min. The splitless injection was used, and the injection port temperature was 280°C. The initial temperature was 50 °C, maintenance time was 2 min, heating rate was 20°C/min, and hold time was 3 min.

*Mass spectrometry conditions*. Ion source and transfer tube temperatures were 250 °C and 285 °C, respectively, and the splitless mode was applied. The electron bombardment ionization mode was used with an ionization energy of 70 eV, a scanning range of 50‒500 amu, and a solvent delay of 6 minutes. The ion scanning acquisition method was adopted, and the ions used for quantitative analysis were at m/z178, 163, 117, and 121 (Propofol) and m/z150, 135, 115, and 90 (thymol). The injection volume was 1 µl, and the mass spectrum data obtained were searched in the National Institute of Standards and Technology General Library of the United States.

*Preparing standard working solution and internal standard solution*: Blank plasma and acetonitrile were used to dilute the Propofol standard solution to 0.02, 0.04, 0.1, 0.2, 0.4, 0.8, 2, 4, 10, and 20 μg_·_mL^-1^ Propofol working solutions, which were stored in a refrigerator at 4°C for subsequent use. Thymol was diluted with acetonitrile to prepare a 500 μg·mL^-1^ thymol acetonitrile working solution, which was stored in a refrigerator at 4°C for subsequent use. The 10 different concentrations of Propofol standard working solution were used to calculate the linear relationship, method specificity, extraction recovery rate, accuracy, and precision.

*Plasma sample pretreatment*: To remove the protein in the collected plasma samples, 200 µl of Propofol plasma sample and 200 µl of thymol acetonitrile working solution were added to a 0.5 m Eppendorf tube, vortexed for 30 s, and centrifuged at 13,000 rpm for 15 min. Subsequently, the supernatant was passed through a membrane. Finally, a single injection volume of 1 µl was used for gas mass spectrometry-chromatographic analysis to determine Propofol concentration in the blood sample.

### 2.3 Calculation of Propofol Pharmacokinetic Parameters

The pharmacokinetic parameter analysis software DAS3.3.1 (Institute of Clinical Pharmacology, Shanghai University of Traditional Chinese Medicine, Shanghai, China) was used to analyze the Propofol plasma samples collected at different altitudes at different time points and calculate the relevant pharmacokinetic parameters, including elimination half-life (t_1/2_), peak plasma concentration (C_max_), area under concentration-time curve (AUC), clearance rate, and mean retention time (MRT).

### 2.4 Statistical Methods

All quantitative data are expressed as mean ± 95% confidence intervals. Data were analyzed using a one-way analysis of variance (ANOVA) using the statistical package for social sciences version 26.0 (IBM, Armonk, NY, USA). The differences between the means of the two groups were compared using the least significant difference post-hoc test. Results were considered statistically significant at *P* < 0.05.

## 3 RESULTS

### 3.1 Baseline Data

No statistically significant differences were observed in the baseline weight data of each group of rats in this study (Table **1**).

### 3.2 Methodology of Gas Chromatography-mass Spectrometry

**Fig. (1):**
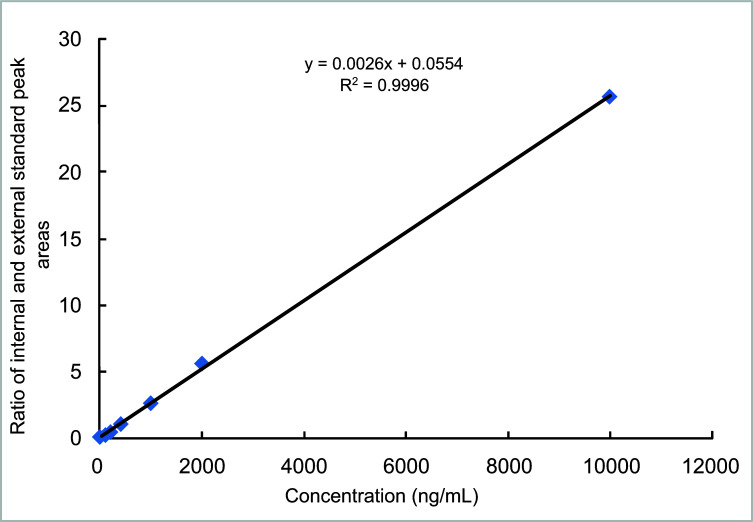
Standard curve.

*Standard curve:* The ratio of the peak area between the external and internal standards was used as the ordinate, and the measured Propofol concentration was used as the abscissa for linear regression analysis. The standard curve equation was y = 0.0026x + 0.0554 (R^2^ = 0.9996). The result indicated that in the concentration range of 0.01-10 μg_·_m^-1,^ the peak area ratio between Propofol and thymol showed an excellent linear relationship with the concentration of Propofol in the plasma (Fig. **1**).

 (The ratio of peak area between the external standard and the internal standard was taken as the ordinate, and the measured concentration of Propofol was taken as the abscissa for linear regression analysis).

#### 3.2.1 Precision and Accuracy

A total of 0.5 m of each of 0.02, 0.4, and 2 μg.m^-1^ Propofol standard working solutions were mixed with 0.5 m of 500 μg·m^-1^ thymol working solution. The mixtures were measured at 8:00 a.m., 14:00, and 20:00 for three consecutive days. The calculated intraday precisions were 6.66%, 2.38%, and 2.58%, and the accuracies were 0.67%, 0.12%, and 3.62%. The calculated interday precisions were 7.15%, 2.99%, and 1.81%, and the accuracies were 2.01%, 0.98%, and 4.72% (Table **2**).

#### 3.2.2 Stability

A total of 0.5 mL of each of 0.02, 0.4, and 2 μg.mL^-1^ Propofol standard working solutions were mixed with 0.5 mL of 500 μg.mL^-1^ thymol working solution. The deproteinized blood samples were placed in a refrigerator at 4°C, room temperature at 25°C, kept frozen at -80°C, and repeatedly frozen and thawed at -80 °C. The samples were then measured and calculated (Table **3**).

#### 3.2.3 Recovery

Low-, medium-, and high-concentration samples were measured using the same method mentioned above, and the recovery results were calculated (Table **4**).

#### 3.2.4 Extraction Recovery Rate

**Fig. (2):**
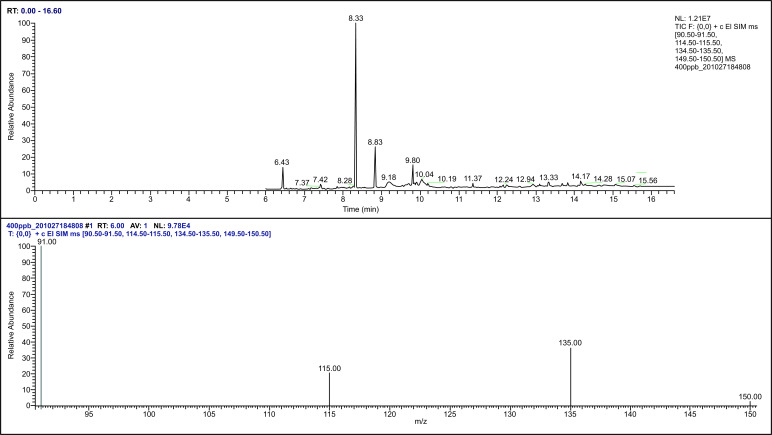
The mass spectrum of thymol.

**Fig. (3):**
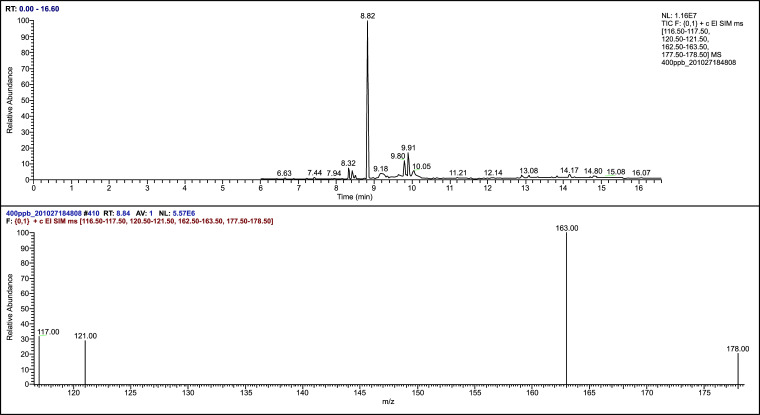
The mass spectrum of Propofol standard.

**Fig. (4):**
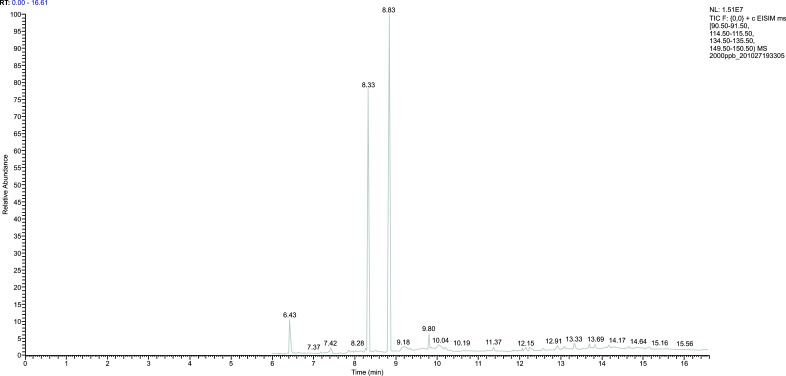
The chromatogram of the internal and external standards.

**Fig. (5):**
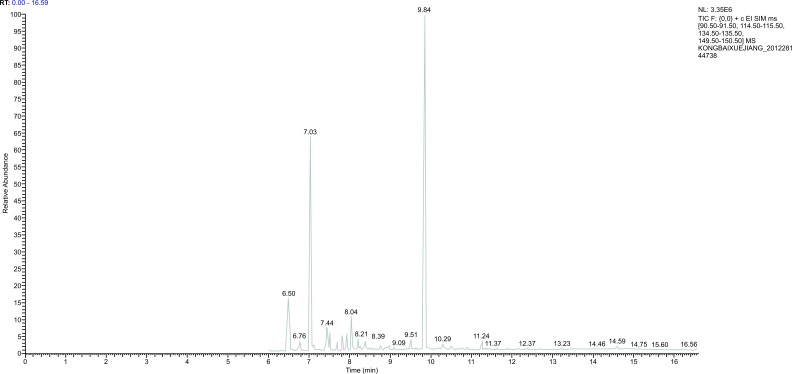
The chromatogram of blank plasma.

**Fig. (6):**
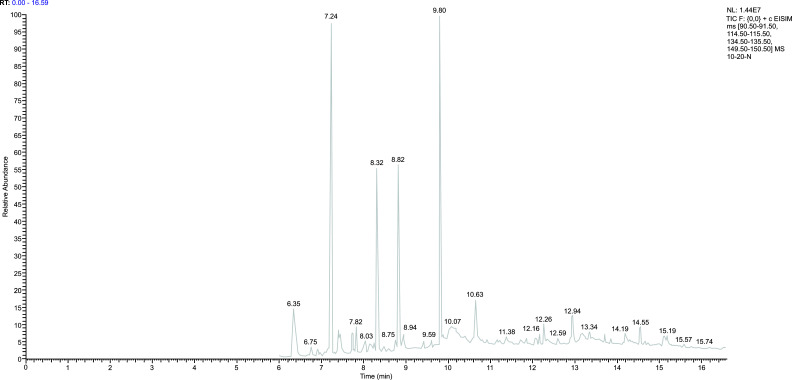
The chromatogram after Propofol infusion for 30 minutes.

After plasma sample pretreatment, blank plasma, Propofol standard solution, internal standard thymol, and plasma samples after Propofol injection through the internal jugular vein were analyzed using gas chromatography-mass spectrometry. The chromatogram revealed that the endogenous substances in the blank plasma did not affect the measured results, and the separation of Propofol and internal standard thymol was significant (Figs. **2**-**6**).

### 3.3 Time for the Disappearance and Recovery of the Righting Reflex

**Fig. (7):**
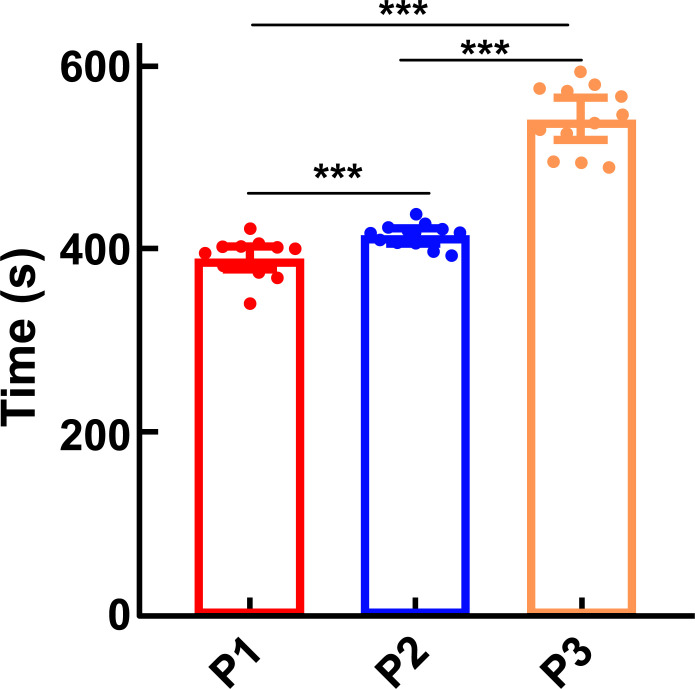
Time of the loss of righting reflex. **Note:** (P1: high-altitude hypoxic group, altitude: 3,900 m, PaO_2_: 12,9 kPa; P2: moderate-altitude hypoxic group altitude: 2,300 m, PaO_2_: 16.1kPa; P3: plain group, altitude: 390 m, PaO_2_: 20kPa; The data are presented as mean ± 95% confidence intervals. n=12. The data were analyzed using ANOVA, and the differences between the means of the two groups were compared using LSD tests. ***p < 0.05 compared to P1 group).

**Fig. (8):**
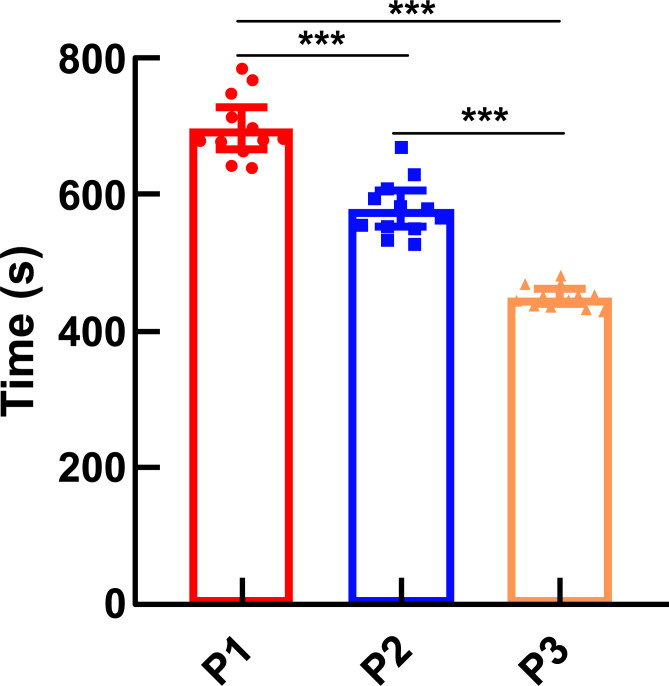
Time of recovering righting reflex. **Note:** (P1: high-altitude hypoxic group, altitude: 3,900 m, PaO_2_: 12,9 kPa; P2: moderate-altitude hypoxic group altitude: 2,300 m, PaO_2_: 16.1 kPa; P3: plain group, altitude: 390 m, PaO_2_: 20 kPa; The data are presented as mean ± 95% confidence intervals. n=12. The data were analyzed using ANOVA, and the differences between the means of the two groups were compared using LSD tests. ***p < 0.05 compared to P1 group).

Under different altitude conditions, each group's time of disappearance and recovery of the righting reflex statistically differed. With increasing altitude, the induction time for Propofol anesthesia became shorter, and the recovery time for anesthesia became longer. Compared with the plain group (541.92 s ± 36.18; 450.50 ± 17.23 s), the moderate-altitude hypoxic (414.75 ± 12.87 s; 578.67 ± 40.89 s) and high-altitude hypoxic (389.50 ± 21.86 s; 679.17 ± 46.57 s) groups showed a shorter time of losing righting reflex and longer recovery time of the righting reflex. Further, compared with the high-altitude hypoxic group, the moderate-altitude hypoxic group had a longer time to lose the righting reflex and a shorter time to recover the righting reflex (Figs. **7** and **8**).

### 3.4 Blood Concentration of Propofol

**Fig. (9):**
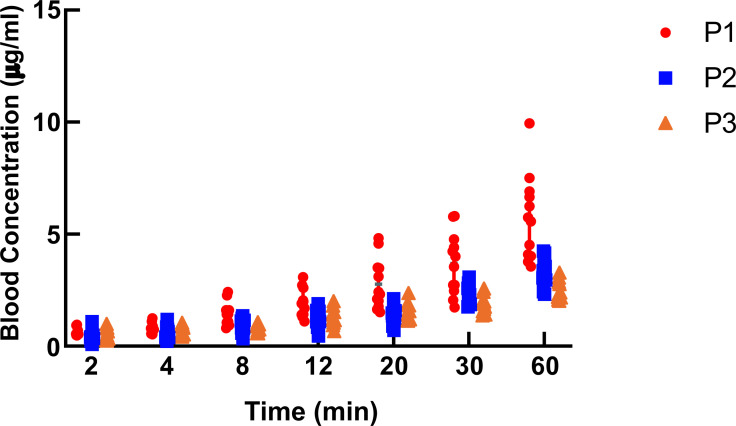
The Propofol plasma concentration at different altitudes and different time points during administration. **Note:** (P1: high-altitude hypoxic group, altitude: 3,900 m, PaO_2_: 12,9 kPa; P2: moderate-altitude hypoxic group altitude: 2,300 m, PaO_2_: 16.1 kPa; P3: plain group, altitude: 390 m, PaO_2_: 20 kPa; The data are presented as mean ± 95% confidence intervals. n = 12).

After continuous intravenous Propofol infusion, the plasma concentrations in the high-altitude group were higher than those in the moderate-altitude hypoxic and plain groups at 8, 12, 20, 30, and 60 min after administration. The plasma concentrations of Propofol at 30 and 60 min after administration in the moderate-altitude hypoxic group were higher than those in the plain group (Table **5** and Fig. **9**). The Propofol concentration in the rats in each group increased with prolonged continuous infusion of Propofol.

**Fig. (10):**
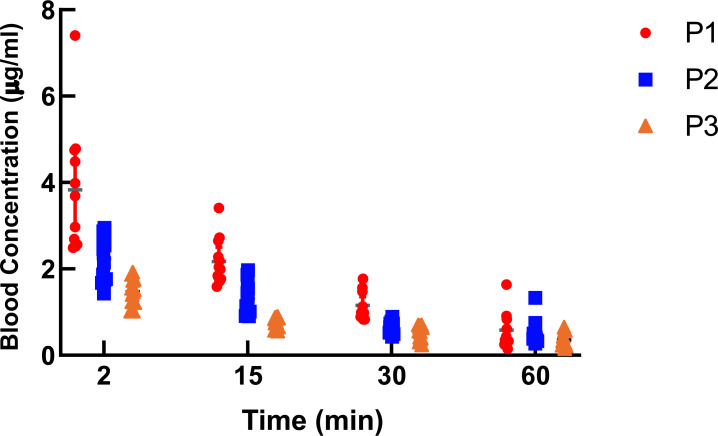
The Propofol plasma concentration at different altitudes and different time points after stopping the drug. **Note:** PaO2: 20 kPa; The data are presented as mean ± 95% confidence intervals. n = 12).

After the discontinuation of Propofol injection, the plasma concentrations of Propofol at 2, 15, and 30 min in the high-altitude group were higher than those in the moderate-altitude hypoxic and plain groups, whereas the plasma concentrations of Propofol at 2, 15, and 30 min in the moderate-altitude hypoxic group were higher than those in the plain group (Table **6**, Fig. **10**). When the infusion was discontinued, the blood concentration of Propofol in the three groups showed a downward trend, and the blood concentration of Propofol in the high-altitude group was higher than that in the middle-altitude and plain groups.

### 3.5 Pharmacokinetic parameters of Propofol

Pharmacokinetic parameters, including C_max_, clearance rate, t_1/2_, AUC, and MRT, were calculated using DAS3.3.1.

Compared with the high-altitude group, the C_max_, t_1/2_, and AUC of the moderate-altitude hypoxic and plain hypoxic groups were lower, and the clearance rates of the moderate-altitude hypoxic and plain groups were higher. The plain group's Cmax, AUC, and MRT were lower than those of the moderate-altitude hypoxic group. Thus, with an increase in altitude, the clearance rate of Propofol in rats decreased, and the t_1/2_, AUC, C_max_, and MRT increased (Table **7**).

## 4 DISCUSSION

Numerous studies have shown changes in drug metabolism under simulated hypoxia [8, 11, 12]; however, differences exist in simulated and real hypoxic environments. Hypoxia models induced by low-pressure cabins or chemical methods can only simulate hypoxia but not hypothermia, dry climate, or intense radiation, indicating that these methods cannot reflect a real high-altitude environment. According to previous reports, hypoxia affects drug metabolism in high-altitude environments, and other factors can affect drug metabolism by altering the expression and activity of the liver-drug metabolism enzymes, drug transporters, hypoxia-inducible factors, and gut microbiota [13]. In this study, animal experiments were conducted in the natural geographic environment at high- (altitude: 3,900 m), medium- (altitude: 2300 m), and low-altitude (altitude: 390 m) regions.

(P1: high-altitude hypoxic group, altitude: 3,900 m, PaO_2_: 12,9 kPa; P2: moderate-altitude hypoxic group altitude: 2,300 m, PaO_2_: 16.1 kPa; P3: plain group, altitude: 390 m, PaO_2_: 20 kPa; The data are presented as mean ± 95% confidence intervals. n = 12. The data were analyzed using ANOVA, and the differences between the means of the two groups were compared using LSD tests. ***p < 0.05 compared to P1 group).

This study conducted a behavioral experiment on the disappearance and recovery of the righting reflex in rats. We found that with increasing altitude, the time of disappearance and recovery of the righting reflex in rats under Propofol anesthesia was shortened and prolonged, respectively. In animal experiments, we used the loss and recovery of the righting reflex as measures to reflect the status of unconsciousness and consciousness, respectively [14, 15]. However, EEG activity, which can reflect the depth of anesthesia, is closely related to the behavior during general anesthesia. In our experiment, we did not monitor EEG changes in rats under Propofol anesthesia at different altitudes. Further research should be conducted to determine whether high-altitude environments affect specific brain regions.

The concentration of Propofol in rat plasma was determined by gas chromatography-mass spectrometry. The results showed that the Propofol concentration in the plasma increased with increasing altitude, which is consistent with our behavioral results. The pharmacokinetic parameters of Propofol at different altitudes showed that with increasing altitude, the clearance rate of Propofol in rats decreased, t_1/2_ was extended, and average residence time, AUC, and C_max_ increased. Researchers have studied the impact of high altitudes on drug metabolism. These results indicate that high-altitude environments can reduce the metabolism of most drugs [3, 16, 17]. However, the mechanisms underlying these changes remain unclear.

Initially, hypoxia was speculated to cause changes in blood flow and plasma protein content in metabolic organs [18]. The liver's uptake rate of Propofol through the blood can be as high as 90%; therefore, it can be seen that the metabolism of Propofol in the liver largely depends on the liver's blood flow and liver function. Any factor leading to reduced liver blood flow greatly affects the metabolism of Propofol [19]. High-altitude hypoxia can reduce liver blood flow and the activity of liver mitochondria, leading to a slower metabolism of Propofol. Moreover, it was found that the expression of drug-metabolizing enzymes, including phase I and II enzymes, could be a major cause. The metabolism of Propofol comprises two phases: Phase I refers to oxidation, reduction, and hydrolysis reactions, and Phase II refers to the combination reaction, which mainly includes glucuronidation, sulfation, and acetylation reactions. The entire process of Phase I requires the participation of oxygen. The enzyme that plays a key role in phase I is the cytochrome P450 enzyme system (CYP450) in the liver, which includes CYP2B6, CYP2C9, CYP1A2, and CYP3A4 [20, 21]. Phase II metabolic processes of Propofol are primarily related to the uridine diphosphate-glucuronosyltransferases (UGT) enzyme family [22, 23]. However, the pharmacokinetics of Propofol metabolites and the content of the metabolic enzymes CYP3A, CYP2C, CYP2B, and UGT have not been determined. Future research should explore the metabolic mechanism of Propofol in high-altitude hypoxic environments, including the regulatory effects of metabolic enzymes such as CYP3A4, CYP2C9, CYP2B6, and UGT.

The mechanism of hypoxia that affects drug metabolism is still unclear. Early evidence indicated that hypoxia redistributes blood in the organs and reduces hepatic blood flow, preventing drug delivery to the liver and thereby decreasing drug eliminations. At present, the study of the mechanism of hypoxia that affects drug-metabolizing enzymes and transporters mainly focuses on correlation factors, nuclear receptors, and correlation signaling pathways [7, 24]. HIF-1 is an important regulator of cellular response to hypoxia, which initiates genetic transcription related to hypoxia. Under normoxic conditions, Proline Hydroxylase (PHD) is activated, and the protease hydrolyzes HIF-1. Under hypoxic conditions, HIF-1 is stabilized by the inhibition of PHD, which further regulates the expression of CYP2C11, CYP3A4, and MDR1[25].The expression of miRNA-18a was significantly decreased in hypoxia with the target mRNA of HIF-1 [26].

The drug absorption, distribution, metabolism, and excretion (ADME) are changed under hypoxic conditions, which alter the pharmacokinetics. Drug transporters play a key role in drug absorption. MiR-873-5p can act on the multidrug resistance protein 1 (MDR1) gene; however, multiple miRNAs can act on the pregnane X receptor (PXR), and the miRNA-PXR-drug transporter axis is important in the physiological disposition of drugs [5]. Propofol is transported to tissue by MDR1, and we can infer that hypoxia affects Propofol redistribution and metabolism. This study is limited to the undetermined Propofol metabolite, which provides a more complete basis. The activity and expression of CYP3A4, CYP2C9, CYP2B6, and UGT need to be detected, and the HIF pathway regulation mechanism needs further research.

Additionally, the pathophysiological changes caused by hypoxia are related to the speed of entering the hypoxic environment and the altitude [27, 28]. We have only studied the effect of chronic hypoxia on the pharmacokinetics of Propofol. Further research should be conducted on the metabolic changes of Propofol under acute hypoxia conditions.

## CONCLUSION

In summary, we investigated the pharmacokinetic changes in Propofol in high-altitude hypoxic environments using animal models and found that the time of disappearance and recovery of the righting reflex decreased and increased, respectively, with increasing altitude. The t_1/2_ of Propofol was prolonged, C_max_ increased, average residence time increased, and clearance rate reduced. Therefore, the metabolism of Propofol in high-altitude hypoxic environments is slow. This study demonstrated the pharmacokinetics of anesthetics in high-altitude areas, which is of great significance for the rational use of anesthetics in high-altitude areas and promotes personalized medication in these areas.

Consideration to reduce the dosage or extend the dosing interval of Propofol at high altitudes to maintain the drug concentration in the therapeutic window since the rate of metabolism and elimination is reduced.

## Figures and Tables

**Table 1:** The body weight of rats (g).

**Group**	**Weight**	**F**	**P**
P1	329.2 ± 8.90	0.788	0.463
P2	332.9 ± 9.83		
P3	333.5 ± 8.00		

**Table 2:** Precision and accuracy.

**Concentration (μg.mL^-1^)**	**Intra-day**			**Inter-day**		
	***x̄* ± s**	**Precision**	**accuracy**	***x̄* ± s**	**Precision**	**accuracy**
0.01	10.067 ± 0.671	0.67	6.66	10.201 ± 0.730	2.01	7.15
0.2	199.765 ± 4.746	0.12	2.38	201.956 ± 6.047	0.98	2.99
1	1036 ± 26.747	3.62	2.58	1047.163 ± 18.920	4.72	1.81

**Table 3:** Stability.

**Concentration (μg.m)**	**25°C**	**4°C**	**Frozen at -80°C**	**Repeatedly frozen and thawed at -80°C**	**RSD(%)**
	*x̄* ± s	*x̄* ± s	*x̄* ± s	*x̄* ± s	
10	10.045 ± 0.595	10.662 ± 0.158	10.064 ± 0.158	9.736 ± 0.607	3.38
200	204.999 ± 1.674	208.657 ± 3.552	199.493 ± 3.579	188.687 ± 3.821	3.91
1000	1067.586 ± 13.564	1076.728 ± 10.667	990.299 ± 12.746	971.997 ± 12.746	7.18

**Table 4:** Recovery.

**Concentration (μg.m)**	**Recovery**	
	***x̄* ± s**	**RSD(%)**
10	112.84% ± 0.038	3.38
200	104.77% ± 0.041	3.91
1000	101.1% ± 0.073	7.18

**Table 5:** The Propofol plasma concentration at different altitudes and different time points during administration (μg^-1^).

**Group Time**	**P1(n = 12)**	**P2(n = 1)**	**P3(n = 12)**	**F**	***P***
T2	0.64 ± 0.16	0.44 ± 0.25	0.49 ± 0.25	32.236	<0.001
T4	0.83 ± 0.20	0.59 ± 0.27	0.67 ±0.21		
T8	1.43 ± 0.51	0.92 ± 0.29^*^	0.82 ± 0.16^*^		
T12	1.93 ± 0.61	1.24 ± 0.39^*^	1.24 ± 0.38^*^		
T20	2.77 ± 1.12	1.38 ± 0.38^*^	1.56 ± 0.5^*^		
T30	3.69 ± 1.37	2.27 ± 0.41^*^	1.78 ± 0.47^*#^		
T60	5.71 ± 1.89	3.32 ± 0.63^*^	2.47 ± 0.41^*#^		
F	141.453				
P	<0.001				

**Note:** compared with P1 group, ^*^p < 0.05; compared with P2 group, ^#^*p* < 0.05.

**Table 6:** The Propofol plasma concentration at different altitudes and different time points after stopping the drug (μg.mL^-1^).

**Group Time**	**P1(n = 12)**	**P2(n = 12)**	**P3(n = 12)**	**F**	***P***
S2	3.83 ± 1.14	2.24 ± 0.53^*^	1.46 ± 0.31^*#^	32.645	<0.001
S15	2.17 ± 0.53	1.31 ± 0.38^*^	0.75 ± 0.13^*#^		
S30	1.15 ± 0.32	0.65 ± 0.13^*^	0.51 ± 0.17^*#^		
S60	0.58 ± 0.41	0.52 ± 0.29	0.35 ± 0.16		
F	152.153			-	-
P	<0.001			-	-

**Note:** compared with P1 group, ^*^p  < 0.05; compared with P2 group, ^#^*p * < 0.05.

**Table 7:** The pharmacokinetic parameters of Propofol.

**Pharmacokinetic Parameters**	**P1(n=12)**	**P2(n=12)**	**P3(n=12)**	**F**	***p***
Cmax (µg/m)	5.71 ± 1.88	3.32 ± 0.63^*^	2.47 ± 0.41^*#^	18.985	<0.001
T½ (min)	32.54 ± 6.79	25.21 ± 5.42^*^	21.97 ± 4.08^*^	11.469	<0.001
CL (m/h.kg)	201.43 ± 52.14	323.78 ± 60.98^*^	366.73 ± 84.15^*^	19.592	<0.001
AUC (0-t)(ug/mL.min)	304.96 ± 79.37	182.30 ± 31.46^*^	141.06 ± 13.91^*#^	17.346	<0.001
AUC (0-∞)(ug/mL.min)	316.60 ± 80.67	191.48 ± 36.24^*^	175.35 ± 58.64^*^	19.114	<0.001
MRT (0-t) (min)	52.00 ± 2.15	52.60 ± 2.43	49.58 ± 0.36^#^	4.653	0.017
MRT (0-∞) (min)	55.72 ± 4.24	57.51 ± 6.74	65.10 ± 7.25	18.4461	0.301

**Note:** compared with P1 group, ^*^p<0.05; compared with P2 group, ^#^*p*<0.05.

## Data Availability

All the data and supporting information is provided within the article.

## LIST OF ABBREVIATIONS

UGT: Uridine Diphosphate-glucuronosyltransferases
PHD: Proline Hydroxylase
SPF: Specific-pathogen-free
MRT: Mean Retention Time
